# New peptide-based and animal-free coatings for animal cell culture in bioreactors

**DOI:** 10.1186/1753-6561-7-S6-P15

**Published:** 2013-12-04

**Authors:** Youlia Serikova, Aurélie Joly, Géraldine Nollevaux, Martin Bousmanne, Wafa Moussa, Jonathan Goffinet, Jean-Christophe Drugmand, Laurent Jeannin, Yves-Jacques Schneider

**Affiliations:** 1 Laboratory of Cellular, Nutritional and Toxicological Biochemistry, Institute of Life Sciences, UCLouvain, 1348 Louvain-la-Neuve, Belgium; 2 Peptisyntha sa, 1120 Brussels, Belgium; 3 ATMI, 1120 Brussels, Belgium

## Background

Anchorage dependent cells require an appropriate extracellular matrix for their survival, migration, proliferation, phenotyping and/or differentiation [1-3]. These cells interact with extracellular matrix proteins, primarily through integrins, which induces focal adhesion contacts assembly and activation of signalling pathways that regulate diverse cellular processes [4].

Culture supports usually include biochemical components allowing such cells to adhere and to reconstitute an extracellular environment close to that found *in vivo*. Currently, this artificial environment is achieved by extracellular matrix constituents deposition, adsorption or grafting; among them collagens, fibronectin, laminin, artificial *lamina propria*... [5]. However, such animal proteins used in cell culture may induce pro-inflammatory stress, be unstable against proteolysis or loose activity after adsorption [6,7]. Synthetic microenvironments should be more suitable for clinical purposes: (i) improved control of physicochemical and mechanical properties, (ii) limited risks of immunogenicity, (iii) increased biosafety (animal free) and (iv) facilitated scale-up [1].

In this framework, research has recently focused on synthetic peptides or peptidomimetics that can mimic the extracellular matrix. Such molecules can be immobilized as recognition motifs on the surface of culture supports with a greater stability and easier surfaces characterization [5]. Self-assembling peptide hydrogels could mimic the chemical and mechanical aspects of the natural extracellular matrix [8,9] by undergoing large deformations, as in mammalian tissues. They have an inherent biocompatibility and should be able to direct cell behaviour [10]. They also can be functionalized with various biologically active ligands constituting good candidates to a new range of smart biomaterials, able to ensure adhesion of different cell types [11-13].

The range of biomimetic peptides that direct cell adhesion and are recognized by integrins is large. Recognition sequences derived from different extracellular matrix proteins include RGD [1], which are specific to different cell lines [1,5,6].

In this context, this work aims at designing animal-free, chemically defined and industrially scalable coatings for animal cell culture, as an alternative to collagen, fibronectin or Matrigel^® ^for laboratory and industrial large scale applications. These are based on self-assembling short peptides bearing adhesion bioactive sequences like RGD-derived or other adhesion sequences developed to coat polystyrene or polyethylene terephthalate surfaces. Adhesion sequences should be recognized by cells, which should favour their anchorage and spreading.

## Experimental

Bioactive self-assembling peptide sequences were synthesized in liquid phase, purified, analytically characterised and manufactured by Peptisyntha (Brussels, Be) in GMP conditions, as sterile coating solutions. They were used to coat polystyrene flasks (Corning Inc., NY) in comparison with animal-derived coatings *i.e*. collagen and fibronectin.

Human Adipose Derived Stem Cells (hADSC) were purchased from Lonza (Verviers, Be); Caco-2, MRC-5 and CHO cells, obtained from ATCC. Cells were seeded at 8 000 cells/cm^2 ^and cultured until 7 days. After 60 h or 7 days of culture, cells were harvested and counted on Bürker cell in Trypan blue or fixed. Nuclei were then stained with DAPI and actin filaments with Rhodamin-Phalloidin. Fluorescence microscopy was used to observe cell morphology and NIS software allowed cell-spreading determination.

## Results and discussion

The absence of cytotoxicity was assayed with necrosis (LDH) and cell metabolic activity (MTT) tests on different cell lines (Caco-2, MRC-5, CHO, hADSC). No cytotoxicity was detected.

Two variants of bioactive self-assembling peptides, both containing RGD-derived sequences, were compared with animal-derived coatings (collagen and fibronectin) in serum-poor of free medium. Cytocompatibility and dose dependent response studies revealed that peptides promote cell adhesion and growth.

As for hADSC culture, these cells were first incubated in a serum-free medium during 6 to 24 h and the proportion of adherent cells and their spreading was evaluated. hADSC cells needed more than 6 h to fully adhere to the culture surface and the adhesion effectiveness appeared better for collagen and the first variant of peptide than for the other substrate coatings. Initial spreading was more marked on fibronectin, but then increased from 6 to 24 hours on all coatings.

**Figure 1:**
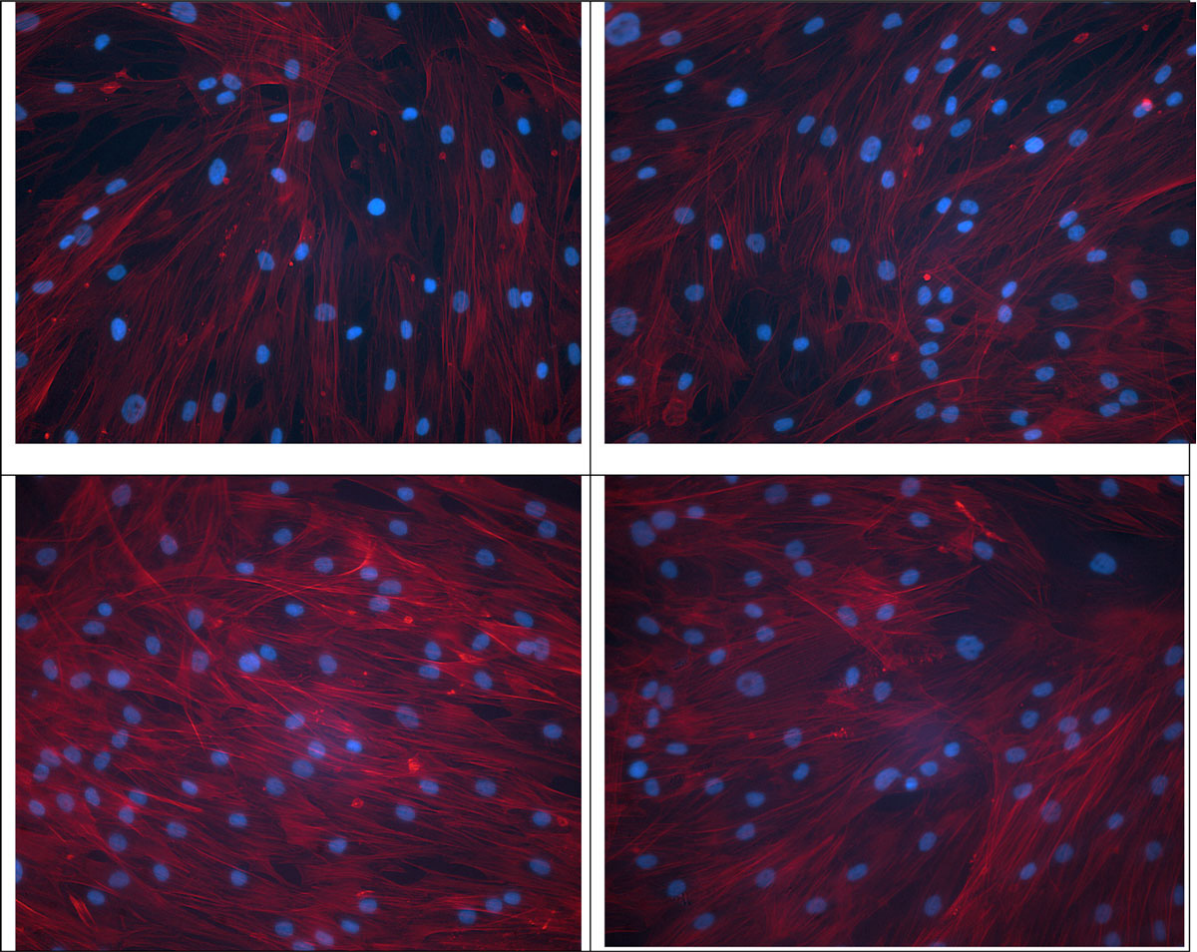
**Fluorescence micrographies of hADSC cultivated for 7 days on polystyrene substrates**. After incubation and cell fixation, nuclei were stained with DAPI and actin filaments with rhodamin-phalloidin. Pictures were taken at the centre of each flask. Upper left: collagen coating; upper right: fibronectin. Lower left: peptide 1; lower right: peptide 2.

A second experiment consisted in a first cell incubation in DMEM supplemented with 1% Fetal Bovine Serum (FBS) and, after 24 h, the medium was replaced by DMEM supplemented with 10% FBS. After 7 days, the best cell growth was observed for substrates coated with collagen and peptide 1, fibronectin and peptide 2 being slightly less efficient. In parallel, cell spreading decreased or remained constant upon cell proliferation (Figure 1).

As for Caco-2 cells culture, these cells were incubated in a serum-free, hormono-defined medium (BDM) during 6 to 24 h and the proportion of adherent cells and their spreading were evaluated. These cells required a shorter duration than hADSC to adhere on the surface and the adhesion effectiveness appeared a little bit better for collagen and fibronectin. Initial spreading was more marked on collagen and its importance varies between 6 and 24 h on different coatings.

The second experiment consisted in a first cell incubation in a serum-free medium and, after 24 h, the nutritive medium was replaced by a medium supplemented with 1% FBS. After 60 h, there was almost no difference between the different coatings. Nevertheless, after 7 days, cells cultured on peptides reached the same effectiveness as on fibronectin, but slightly lower than collagen. As for hADSC, cell spreading decreased upon cells proliferation.

## Conclusion

Designed self-assembling bioactive peptides are not cytotoxic and are cytocompatible. Cell adhesion and growth on peptide coatings appear as effective as on animal-derived coatings and the peptide coatings allow easy cell harvesting after culture.

Globally, the results indicate that self-assembling bioactive peptides constitute chemically defined, entirely synthetic and effective promoters of cell adhesion, spreading and proliferation.
